# Influence of lactulose as a composition of organic-mineral feed additive on broiler chicken productivity, feed digestibility, and microbiome

**DOI:** 10.14202/vetworld.2025.2095-2105

**Published:** 2025-07-30

**Authors:** Elena A. Sizova, Daniil E. Shoshin, Elena V. Yausheva, Anastasia P. Ivanishcheva, Ksenia S. Nechitailo, Kristina V. Ryazantseva

**Affiliations:** 1 Federal Research Centre of Biological Systems and Agrotechnologies of the Russian Academy of Sciences, 29, 9 Yanvarya Street, Orenburg, 460000, Russia; 2 Scientific Educational Center “Biological Systems and Nanotechnologies”, Orenburg State University, 13, Pobedy Avenue, Orenburg, 460018, Russia

**Keywords:** arginine, broiler chickens, digestibility, feed additive, lactulose, microbiome, silicon dioxide, succinic acid

## Abstract

**Background and Aim:**

The global demand for efficient poultry production necessitates alternatives to antibiotic growth promoters. This study aimed to evaluate the effects of a novel four-component organic-mineral feed additive (OMFA), comprising lactulose, arginine, ultrafine silicon dioxide particles, and succinic acid, and a three-component variant (without lactulose) on growth performance, nutrient digestibility, elemental tissue composition, and the cecal microbiota of Arbor Acres broiler chickens.

**Materials and Methods:**

One hundred and five one-day-old broiler chicks were randomly allocated into three groups: Control, Group I (four-component OMFA), and Group II (three-component OMFA). Growth metrics were recorded weekly over a 42-day period. Nutrient digestibility was assessed through balance experiments, while elemental tissue composition was measured by inductively coupled plasma mass spectrometry. Cecal microbiota profiling was conducted using 16S *ribosomal RNA* gene sequencing on the MiSeq platform. Statistical analyses were performed using the Mann-Whitney U-test.

**Results:**

Group I showed an 11.2% increase in body weight gain and a 9.6% reduction in feed conversion ratio compared to controls (p = 0.074; p = 0.063). Group II demonstrated superior weight gain (17.9%) but incurred a 3.6% increase in feed costs. Digestibility of crude fat and protein improved significantly in Group II (p = 0.037). Elemental analysis indicated that lactulose supplementation enhanced the accumulation of magnesium, calcium, manganese, cobalt, zinc, and chromium in muscle tissue. Microbiota analysis revealed that Group I increased *Ruminococcaceae* abundance and suppressed *Pseudobdellovibrionaceae*, while Group II favored the proliferation of *Helicobacteraceae, Rikenellaceae*, and *Bacteroidaceae.*

**Conclusion:**

Both OMFA formulations enhanced productivity and modulated gut microbiota. The four-component OMFA improved feed efficiency and mineral deposition, while the three-component version elicited greater weight gains. These findings support the incorporation of OMFA as a strategic tool in antibiotic-free poultry production. Further studies are warranted to elucidate the metabolic interactions among additive components and their long-term effects on gut health and performance.

## INTRODUCTION

To satisfy the growing global demand for animal-derived protein, poultry production has experienced a significant increase, largely driven by advancements in nutrition, genetics, and management strategies [1–3]. This expansion is attributable to the rapid evolution of the poultry industry, where modern technologies now permit the production of market-ready carcasses within 35–42 days. Such accelerated growth is achieved not solely through the provision of a balanced diet but also through the incorporation of feed additives with diverse functional properties [4, 5]. Among these, prebiotics have emerged as a particularly promising class of additives. Unlike antibiotics, prebiotics do not adversely affect the safety and quality of animal products intended for human consumption [6]. Their beneficial role is primarily due to their capacity to promote competitive exclusion of pathogenic microorganisms by selectively enhancing the metabolic activity and proliferation of beneficial gastrointestinal microflora. Consequently, prebiotics improve the overall physiological status and productivity of poultry while mitigating the risks associated with antimicrobial resistance development and antibiotic transmission through the food chain [7, 8].

In light of this context, the identification and validation of high-potential prebiotic agents remain a research priority. Among such agents, oligosaccharides, especially lactulose, a synthetic structural isomer of lactose, have shown considerable promise. Lactulose is distinguished by its potent prebiotic activity, which promotes the growth of beneficial lactobacilli and bifidobacteria in the large intestine, restore healthy microbiota, reduces intestinal pH, inhibits the growth of opportunistic pathogens, enhances nutrient absorption, and strengthens immunity [9, 10]. Nonetheless, uncertainties persist regarding its sustained efficacy, particularly when used in combination with mineral complexes of varied composition.

The formulation of the organic-mineral feed additives (OMFAs) was mentioned by previous studies. Besides lactulose, the additive includes ultrafine SiO_2_ particles, recognized for their immunomodulatory properties [11–13]; succinic acid, which acts as an acidifier and enhances the body’s defense mechanisms [14, 15]; and arginine, which regulates carbohydrate metabolism and serves as a precursor for creatine and creatinine synthesis. Arginine is essential for growth and reproductive functions, is linked to parathyroid activity, and contributes to the production of the arginase enzyme [16, 17]. This experimental design enables an evaluation of potential synergistic or antagonistic interactions among these functionally diverse components, thereby informing strategies for either combined or sequential administration.

Despite the growing body of literature supporting the beneficial effects of prebiotics such as lactulose on poultry growth performance, nutrient utilization, and gut microbiota modulation, several knowledge gaps remain. Most existing studies have evalua-ted lactulose in isolation or in simple formulations, overlooking its potential synergistic or antagonistic interactions with other functional dietary components. The sustained efficacy of lactulose when integrated into complex OMFAs, particularly in conjunction with immunomodulatory agents, such as ultrafine silicon dioxide, energy substrates like succinic acid, and essential amino acids such as arginine, remains poorly understood. Furthermore, limited evidence is available on the effects of such composite formulations on the elemental composition of broiler muscle tissue and the structure of the cecal microbiota. The temporal dynamics of microbial shifts in response to these additives, as well as their implications for feed conversion efficiency and metabolic enhancement, require further elucidation.

This study was conducted to evaluate the efficacy of a novel four-component OMFA, comprising lactulose, arginine, ultrafine silicon dioxide particles, and succinic acid, compared to a three-component formulation lacking lactulose, in Arbor Acres broiler chickens. Specifically, the research aimed to assess and compare the impact of these formulations on growth performance, feed digestibility, elemental deposition in muscle tissue, and the composition of the cecal microbiota. The inclusion of functionally diverse bioactive compounds was intended to investigate their potential interactions and combined effects on metabolic efficiency, microbiome modulation, and overall poultry productivity under controlled conditions.

## MATERIALS AND METHODS

### Ethical approval

All procedures involving animals were conducted in accordance with the Guide for the Care and Use of Laboratory Animals and ARRIVE guidelines (National Research Council, US Institute for Laboratory Animal Research, 2016). The experimental protocol was approved by the Ethics Committee of the Federal Research Center of Biological Systems and Agrotechnologies of the Russian Academy of Sciences (Protocol No. 1, March 22, 2021).

### Study period and location

The study was conducted from July 2021 to August 2022, utilizing the instrumentation facilities of the Center for Collective Use of Biological Systems and Agricultural Technologies of the Russian Academy of Sciences (http://tskp-bst.rf).

### Animals and experimental design

The experiment was conducted on Arbor Acres broiler chickens housed in a vivarium. A total of 105 one-day-old chicks were individually tagged and weighed, then maintained under identical conditions. At 2 weeks of age, based on feed intake and individual weight gain, the birds were allocated into three groups (n = 35 per group) using the analog pair method:

- Control group: Basal diet only
- Group I: Basal diet + four-component OMFA (lactulose, arginine, ultrafine silicon dioxide, and succinic acid)
- Group II: Basal diet + three-component OMFA (same as Group I, without lactulose).

The composition and dosage of the additives were determined based on previous research by Ivanishcheva *et al*. [18] and Ivanishcheva and Sizova [19].

### Diets and feeding regimen

Broilers were fed nutritionally balanced com-pound feeds formulated according to age-specific requirements, following standardized protocols [20]. The feed composition varied by age phase:

- 0–10 days (g/kg): Wheat 380, corn 150, soybean meal 200, sunflower meal 150, sunflower oil 50, table salt 1, monocalcium phosphate 14, limestone flour 5, DL-methionine (98.5%) 1, L-threonine (98%) 5, and baking soda 1
- 11–28 days: Wheat 465, corn 78, soybean meal 250, sunflower meal 70, sunflower oil 50, table salt 3.4, monocalcium phosphate 13, limestone flour 5, DL-methionine 1.6, L-threonine 5, and baking soda 1
- 29–42 days: Wheat 425, corn 220, soybean meal 150, sunflower meal 100, sunflower oil 50, table salt 3, monocalcium phosphate 10, limestone flour 1, DL-methionine 1.2, L-threonine 3.5, and baking soda 1.

### Composition of feed additives (OMFA)

- Lactulose (99%) (Lactomin.ru, Moscow): 1 g/kg of feed; white powder, water-soluble
- Arginine (Lactomin.ru, Moscow): 0.7 g/kg; salt of arginine and alpha-ketoglutaric acid, white bitter powder
- Ultrafine silicon dioxide (SiO_2_) (Highly Pure Substances, Moscow): 0.65 g/kg; odorless white powder, and 96% purity
- Succinic acid (100ING.RU, Moscow): 0.1 g/kg; colorless, sour-tasting crystals, and water- and alcohol-soluble.

### Growth monitoring and performance parameters

Growth was monitored weekly by individual weighing to calculate average daily gain. The trial lasted 42 days. Feed intake and the health status of the birds were monitored daily. The European productivity index (EPI) was calculated as: EPI = (Live weight × Survival rate)/(Fattening days × Feed conversion) × 100%.

### Digestibility trial

Digestibility was evaluated between days 23 and 27 using the FSC ARRTPI (Federal State Budget Scientific Institution Federal Scientific Center “All-Russian Research and Technological Poultry Institute) methodology [21]. Birds were housed in mesh-floor cages with plastic-lined trays for the collection of feces. Droppings were collected and weighed daily; 10% of the total mass was preserved in 0.1 N oxalic acid and stored at 4°C for analysis.

### Sample collection and chemical analysis

Muscle samples (breast) were obtained post-slaughter. Feed, excreta, muscle, and bone samples were analyzed for:

- Dry matter (DM): Oven drying to constant weight
- Crude protein (CP): Kjeldahl method
- Crude fat (CF): Soxhlet ether extraction
- Crude fiber (CFB): Henneberg and Stohmann method
- Carbohydrates (Carb): Anthrone reagent assay
- Nitrogen-free extractive substances (NFS): NFS = Carb – CFB
- Ash: Combustion at 550 ± 25°C.

### Elemental analysis of muscle tissue

Elemental composition (Na, K, Ca, Mg, Mn, Co, Ni, Cu, Fe, Zn, Se, and Cr) was quantified through inductively coupled plasma mass spectrometry (ICP-MS) (ICP-MS; Agilent 7900, California, USA).

### Cecal microbiota collection and analysis

Cecal content was aseptically collected during slaughter using sterile syringes and preserved in microtubes for analysis.

#### DNA extraction and sequencing

Total microbial DNA was extracted using the QIAamp Fast DNA Stool Mini Kit (Qiagen, Germany) after homogenization with a TissueLyser LT and Lysis Matrix Y. DNA quality was verified through 1% agarose gel electrophoresis and NanoDrop 8,000 (Thermo Fisher Scientific, USA), and concentration was measured with a Qubit 4 fluorometer using the dsDNA High Sensitivity Assay Kit (Thermo Fisher Scientific).

DNA libraries were prepared according to Illumina protocols (#15044223, Rev. B; Illumina, USA) using primers for the V3–V4 regions of the *16S ribosomal RNA* (*16S rRNA*) gene (S-D-Bact-0341-b-S-17 and S-D-Bact-0785-a-A-21) [22]. Amplification involved 25 polymerase chain reaction cycles (95°C for 3 min; 95°C, 56°C, and 72°C for 30 s each; final extension at 72°C for 5 min). Purified products were validated by capillary electrophoresis on the QIAxcel system (Qiagen, Hilden, Germany) with the QIAxcel DNA Screening Kit and sequenced using 2 × 251 bp paired-end runs on the Illumina MiSeq platform (reagent kit v.2, Illumina, USA).

### Bioinformatics analysis

Raw reads were quality-checked using FastQC v0.11.7 [23]. Paired reads were merged with USEARCH v10.0.240 using -fastq_mergepairs (maxDiffs 10, pctid 80). Reads were trimmed, re-evaluated, filtered (expected error <1.00, ≥420 bp), dereplicated, and clustered into operational taxonomic units (OTUs) using the UPARSE algorithm [24]. Chimeras were removed with UCHIME2 [25]. OTUs were mapped to the original reads using usearch_global at a 97% identity threshold, and contaminant OTUs were excluded using usearch_ublast. Taxonomic classification was performed using the Ribosomal Database Project reference database [26].

### Statistical analysis

Data were analyzed using Statistica 10.0 (StatSoft Inc., USA) and Microsoft Excel. Results are presented as mean ± standard error (M ± m). Group compar-isons were evaluated using the nonparametric Mann–Whitney U-test. Statistical significance was considered at p ≤ 0.05.

## RESULTS

### Feed consumption and body weight

Throughout the experimental period, livestock survival remained at 100%, indicating that the tested feed additives had no adverse effects. Administration of the four-component OMFA (Group I) resulted in a live weight gain of 2.3 kg, which was 11.2% higher than that of the control group (p = 0.074), alongside a 9.6% reduction in feed consumption per kilogram of gain (p = 0.063) (Table 1). Notably, supplementation with the three-component OMFA (Group II), which excluded lactulose, further enhanced weight gain to 2.4 kg compared to 2.1 kg in the control group (p = 0.011), although this was accompanied by a modest increase in feed intake.

**Table 1:** Live weight gain and feed consumption at the end of the experiment, kg (mean values ± standard error of the mean, M ± m).

Group	Liver weight gain (kg)	Feed consumption per 1 kg of live weight, kg
Control	2.06 ± 0.06	1.87 ± 0.10
I	2.29 ± 0.07	1.69 ± 0.08
II	2.43 ± 0.08	1.75 ± 0.10

Significant difference compared with the control group: *p ≤ 0.05

The EPI also reflected the benefits of OMFA supple-mentation. While the control group had a baseline EPI of 238%, Groups I and II achieved values of 296.1% and 304.8%, respectively, indicating superior productivity with both formulations, particularly with the three-component version.

An evaluation of growth dynamics (Table 2) further supported these findings. During the 1^st^ week, Group I outperformed the control by 18.6% (p = 0.096). However, this advantage diminished by day 28 during the transition to a growth-phase diet, after which both experimental groups exhibited accelerated growth rates compared to the control.

**Table 2:** Dynamics of live weight of broiler chickens in the experiment, g (mean values ± standard error of the mean, M ± m).

Group	Age, days					

	7	14	21	28	35	42
Control	183.3 ± 5.7	309 ± 22.0	497.1 ± 33.9	946.9 ± 61.2	1461.1 ± 72.7	2061.1 ± 61.9
I	182.9 ± 5.6	366.6 ± 15.1	569.7 ± 24.1	906.4 ± 19.6	1525.4 ± 26.1	2285.6 ± 70.7
II	182.7 ± 4.2	353.8 ± 15.7	570.7 ± 20.2	1003.7 ± 40.9	1618.0 ± 60.1	2431.4 ± 83.1*

Significant difference compared with the control group:

* p ≤ 0.05

### Digestibility of feed nutrients

The introduction of OMFA had a notable effect on nutrient digestibility in both starter and growth phases (Tables 3 and 4). The three-component OMFA (Group II) reduced the digestibility of nitrogen-free extractive substances by 12.1% (p = 0.012). Conversely, it significantly improved the digestibility of CF and CP by 8.3% and 4.4%, respectively (p = 0.037 for both). Comparable improvements in fat and protein digestibility were also observed in Group I, although differences for other parameters were not statistically significant.

**Table 3:** Digestibility coefficient of feed nutrients in the starter diet.

Group	Feed nutrients						

	DM	OM	CF	CP	CF	NFS	Carb
Control	71.2 ± 1.5	73.3 ± 1.4	69.2 ± 1.6	77.6 ± 1.2	5.2 ± 4.1	75.3 ± 1.3	72.1 ± 1.5
I	69.7 ± 1.3	70.7 ± 1.3	75.1 ± 1.1	77.2 ± 1	9.9 ± 5.3	70.8 ± 1.3	67.9 ± 1.4
II	73.9 ± 1.2	75.1 ± 1.1	67.5 ± 1.5	79.9 ± 0.9	9.6 ± 3.5	66.2 ± 1.5*	73.8 ± 1.2

DM=Dry matter, OM=Organic matter, CF=Crude fat, CP=Crude protein, CF=Crude fiber, NFS=Nitrogen-free extractive substances, Carb=Carbohydrates; % (mean values ± standard error of the mean, M ± m). Significant difference in relation to control:

* p ≤ 0.05

**Table 4:** Digestibility coefficient of feed nutrients in the growth diet.

Group	Feed nutrients						

	DM	OM	CF	CP	CF	NFS	Carb
Control	80.1 ± 1.7	80.3 ± 1.7	79.6 ± 1.7	85.1 ± 1.3	14.3 ± 7.3	81 ± 1.6	78.8 ± 1.8
I	84.7 ± 0.7	84.7 ± 0.7	83.4 ± 0.7	88.7 ± 0.5*	5.5 ± 4.1	85.4 ± 0.6	83.5 ± 0.7
II	83.9 ± 1.3	83.7 ± 1.3	86.2 ± 1.1	88.8 ± 0.9*	4.2 ± 2.9*	83.9 ± 1.3	81.7 ± 1.5

DM=Dry matter, OM=Organic matter, CF=Crude fat, CP=Crude protein, CFB=Crude fiber, NFS=Nitrogen-free extractive substances, Carb=Carbohydrates, % (mean values ± standard error of the mean, M ± m). Significant difference compared with the control group:

* p ≤ 0.05

In both experimental groups, the digestib-ility of most feed components increased during the growth phase, suggesting a positive impact of OMFA formulations during active rearing. These findings collectively indicate a beneficial effect of OMFA, particularly in the second phase of broiler development when growth demands are highest.

### Chemical composition of broiler chicken tissues

No statistically significant differences were obs-erved in the proximate composition of muscles and bones across groups (Table 5). However, Group I showed favorable trends, including a 2.2% increase in DM and a 6.1% increase in CP content in muscle tissue (p = 0.081), accompanied by an 18.7% decrease in CF (p = 0.081). In both experimental groups, bone tissue exhibited reduced DM and CF content, but a relative increase in protein content.

**Table 5:** Chemical composition of some tissues of broiler chickens at the end of the experiment.

Group	Carcass flesh				Minced meat and bones			
	
	DM	Moisture	CF	CP	DM	Moisture	CF	CP
Control	24.7 ± 0.1	75.3 ± 0.1	5.1 ± 0.3	18.4 ± 0.2	35.3 ± 0.2	64.7 ± 0.2	10.7 ± 0.5	17.5 ± 0.2
I	25.3 ± 0.3	74.7 ± 0.3	4.1 ± 0.3	19.6 ± 0.4	33.6 ± 0.4	66.4 ± 0.4	9.4 ± 0.6	18.4 ± 0.5
II	24.7 ± 0.1	75.3 ± 0.1	4.3 ± 0.3	19 ± 0.2	32.2 ± 0.7	67.8 ± 0.7	8.5 ± 0.7	19 ± 0.7

DM=Dry matter, CF=Crude fat, CP=Crude protein; % (mean values ± standard error of the mean, M ± m). Significant difference compared with the control group: *p ≤ 0.05, **p ≤ 0.01

**Figure 1:**
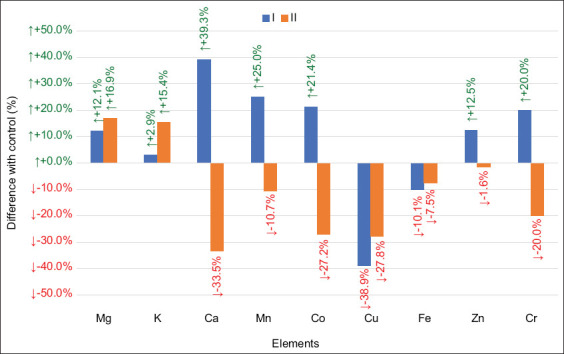
Difference in the concentration of chemical elements in the muscle tissue of broiler chickens between the experimental groups and the control at the end of the experiment, %.

Notably, OMFA supplementation had a significant impact on the mineral composition of muscle tissue (Figure 1). The inclusion of lactulose (Group I) promoted the accumulation of essential macro- and microelements – magnesium, calcium, manganese, cobalt, zinc, and chromium (p = 0.081) – while reducing copper and iron concentrations. In contrast, Group II exhibited decreased concentrations of most minerals, except for magnesium and potassium, which remained relatively stable.

### Cecal microbiota analysis

Microbiota profiling revealed substantial taxonomic diversity across all groups. A total of 738,109 reads were generated from cecal samples, ranging from 71,645 to 92,261 per sample. After merging, filtering, and removing singletons and rare OTUs, 438,708 high-quality reads were retained, representing 1,633 OTUs. These were taxonomically assigned to 7 phyla, with variation in class, order, family, and genus counts across groups: 93 genera in the control, 94 in Group I, and 95 in Group II.

**Figure 2:**
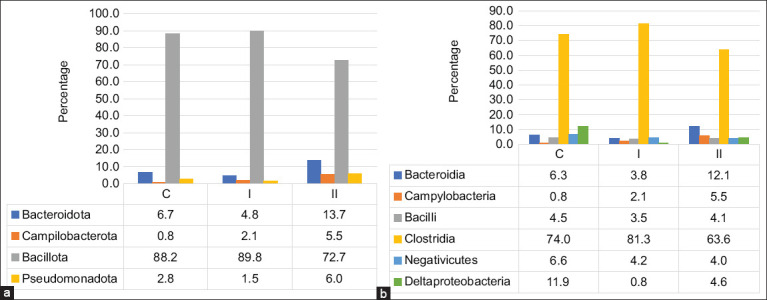
Relative content of the main taxonomic groups of the microbiota of the cecum of broiler chickens at the level of phylum (a) and class (b) at the end of the experiment.

In the control group, 88% of sequences belonged to the phylum Bacillota (Figures 2a and b), predomi- nantly of the class Clostridia (74%). Major families included *Ruminococcaceae* (31.6%), Lachnospiraceae (15.5%), and Clostridiaceae (4.46%). Minor representation was noted for Bacteroidota (6.66%) and Pseudomonadota (2.78%), with lower abundances of Bacteroidia, Bacilli, and Negativicutes. At the genus level, prominent taxa included Mediterraneibacter (10.6%), unclassified Lachnospiraceae (15.5%), unclassi-fied Oscillospiraceae (11.9%), Alistipes (5.28%), Faecali-bacterium (6.65%), Ruminococcus (4.58%), Allisonella (4.22%), and Clostridium (4.46%).

**Figure 3:**
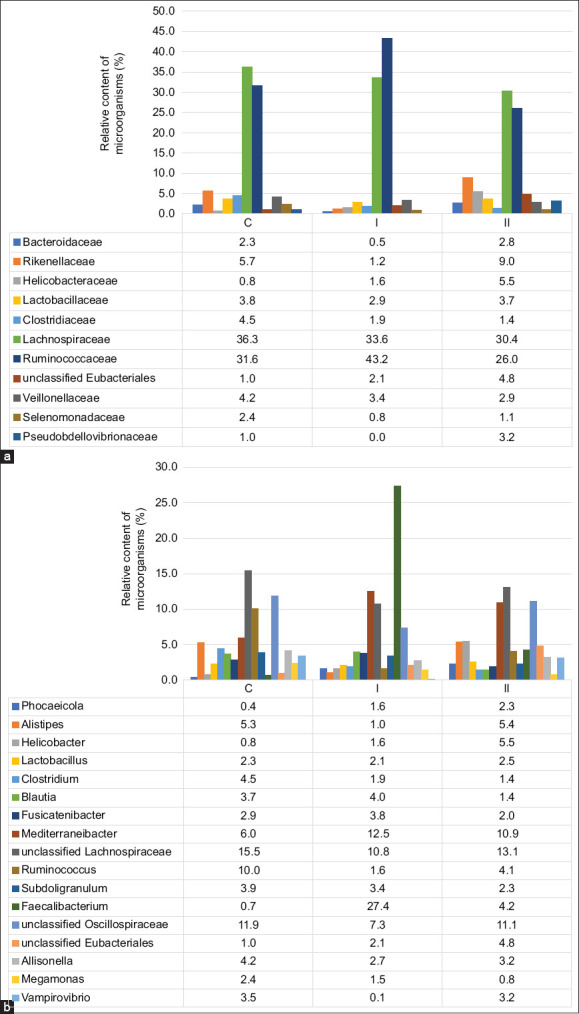
Relative content of the main taxonomic groups of the microbiota of the cecum of broiler chickens at the level of (a) family and (b) genus.

In Group I (OMFA with lactulose), phylum-level distribution remained largely unchanged. However, notable taxonomic shifts were observed at lower levels: Reductions in Bacteroidia (−2.51%), Negativicutes (−12.3%), and Deltaproteobacteria (−159.6%, p = 0.081), alongside a 7.3% increase in Clostridia. Importantly, the relative abundance of *Ruminococcaceae* increased by 11.6%, while *Pseudobdellovibrionaceae* nearly disappeared (p = 0.081). A slight decrease (4%–5%) was observed in Alistipes, unclassified Lachnospiraceae, and Oscillospiraceae (Figures 3a and b).

In contrast, Group II (OMFA without lactul-ose) showed increased representation of the phyla Bacteroidota (+7.09%), Pseudomonadota (+4.7%), and Campylobacterota (+3.2%) compared to the control. This was accompanied by elevated lev-els of *Helicobacteraceae* (+4.68%), *Rikenellaceae* (+3.29%), *Pseudobdellovibrionaceae* (+2.16%), and *Bacteroidaceae* (+2.15%). Conversely, the abundance of Bacillota decreased by 15.5%, particularly in the class Clostridia (−10.4%, p = 0.081) and Negativicutes (−12.5%). Despite the overall decline in Clostridia, there was a 14.9% increase in Lachnospiraceae, suggesting compensatory shifts within the phylum.

## DISCUSSION

Prebiotics, most commonly defined as spe-cific chemical compounds or indigestible feed ingre-dients [27], are metabolized by certain members of the intestinal microbiota. This selective fermentation modulates microbial composition, thereby fostering optimal conditions for the growth and function of commensal flora. These beneficial microbes influ-ence both luminal and mucosal digestive proc-esses, vitamin synthesis, intestinal motility [28], and the production of short-chain fatty acids (SCFAs). SCFAs serve as a key energy source for intestinal epithelial cells and as substrates for the synthesis of sugars and lipids. Additionally, they regulate innate and adaptive immune responses by modulating the activity of macrophages, neutrophils, and dendritic cells, and promoting the differentiation of T and B lymphocytes [29].

The mechanisms underlying the effects of prebiotics, particularly fermentable oligosaccharides derived from fructose, mannose, inulin, xylose, and lactose, extend beyond simple nutrient provision for prokaryotes and protozoa. They also include the prevention of pathogen adhesion, competitive exclusion, antimicrobial compound production, and morphological changes in the intestinal villi, all of which occur in concert with the host immune system [30]. These effects align with previous findings by Gaggìa *et al*. [31] and Patterson and Burkholder [32] demonstrating that OMFA enhances physiological status and growth performance by improving nutrient absorption and metabolic efficiency in broiler chickens.

Previous studies have reported that dietary inclusion of lactulose at levels of 0.15%–0.8% enhances absolute body weight gain, breast muscle yield [33], and nutrient digestibility while reducing the feed conversion ratio [34]. These outcomes are consistent with the current findings on growth dynamics and nutrient utilization in birds receiving the lactulose-containing OMFA (Group I). These benefits are attributed to the ability of oligosaccharides, resistant to digestive enzymes, to be metabolized by cecal bacteria such as *Lactobacillus*, *Bifidobacterium*, and *Bacteroides*, leading to SCFA production [35]. However, in this study, no significant increase in these bacterial genera was observed. Rather, a decline was noted in *Lactobacillus*, *Ruminococcus*, and *Clostridium* – taxa typically associated with high productivity.

Conversely, the abundance of *Mediterraneibacter* and *Faecalibacterium* – belonging to the families *Lachnospiraceae* and *Ruminococcaceae*, respectively – increased significantly. These taxa are known butyrate producers [36], a SCFA with growth-promoting effects that enhances antimicrobial peptide production [37], attenuates inflammation through cytokine modulation [38], inhibits *Salmonella enteritidis* colonization [39], and stimulates epithelial cell proliferation [40]. In addition, ele-vated SCFA concentrations reduce intestinal pH, suppress acid-sensitive pathogens, and enhance mineral absorption [41], which may explain the increased macro- and microelement deposition in muscle tissue observed in Group I (Figure 1).

Cumulatively, a previous study by Zhao *et al*. [34] has confirmed the prebiotic efficacy of lactulose in promoting the growth of *Bifidobacterium* and *Lactobacillus*, while suppressing the populations of *Clostridium*, *Salmonella*, and *Escherichia coli* in the gastrointestinal tract. Thus, the inclusion of lactulose in broiler diets contributes to improved productivity. However, this effect appears to be contingent on the timing of supplementation, potentially related to the immature state of the gut microbiome in early life and the low abundance of functionally active *Bacil-lota*, including *Lachnospiraceae*, *Ruminococcaceae*, and *Clostridiaceae* [42]. Nonetheless, the long-term production benefits and reduced feed costs associated with lactulose-containing OMFA underscore its practical relevance.

In contrast, the three-component OMFA (excluding lactulose) produced the greatest increase in live weight. However, it was associated with higher feed costs per kilogram of weight gain compared to the lactulose-supplemented group. This improved growth may be attributed primarily to the anabolic effects of arginine. Since poultry lack a complete urea cycle and key enzymes such as ornithine carbamoyltransferase and hepatic arginase [43], endogenous arginine synthesis is not possible. Therefore, dietary arginine becomes a limiting nutrient for development, playing critical roles in protein synthesis, polyamine production (through the ornithine pathway), and nitric oxide generation. These metabolites are vital for cellular replication, neurotransmission, and immune modulation [44]. Arginine also stimulates the secretion of insulin, insulin-like growth factor, and somatotropin [45], which collectively enhance growth and feed conversion [46].

Succinic acid and ultrafine silicon dioxide also contributed to growth performance. Succinic acid is a key intermediate in the tricarboxylic acid cycle, which not only supports adenosine triphosphoric acid (ATP) production but also generates metabolic precursors, including oxaloacetate, malate, succinyl-CoA, 2-oxoglutarate, and citrate. These compounds are subsequently converted into glucose, porphyrins, amino acids, fatty acids, and isoprenoids [47], thereby promoting anabolic activity. For instance, Krotova *et al*. demonstrated that succinic acid improved lipid and energy metabolism in *Ross-308* broilers, increased ATP content, and activated ATP synthase, resulting in improved survival and body weight [48].

Silicon, particularly in the form of ultrafine SiO_2_, is essential for connective tissue development, especially bone formation [49]. Its inclusion in poultry diets has been associated with a reduced intestinal pathogen load, improved hematological and biochemical indices (including erythrocytes, hemoglobin, total protein, and albumin), enhanced live weight dynamics, increased nutrient digestibility, and improved energy utilization [50, 51]. However, silicon may also function as an intestinal adsorbent, potentially limiting mineral absorption [52, 53].

It is plausible that lactulose may interact antagonistically with one or more of these three components or compete for membrane transporters during early development, thereby diminishing the overall anabolic effect. Nevertheless, the elevated mineral concentrations observed in the muscle tissue of birds in Group I may be attributed to lactulose-induced improvements in intestinal morphology. A previous histomorphological study by Elkomy *et al*. [54] demonstrated that lactulose increases villus height, goblet cell density, and mucus layer coverage, while deepening the crypts that house digestive enzyme-producing cells and enhancing the muscularis layer responsible for peristalsis and absorption. Conversely, ultrafine SiO_2_ has been reported to damage the brush border membrane by reducing microvilli density, thereby limiting the absorptive surface area [55].

In summary, the three-component OMFA appears to enhance anabolic processes, sustain high metabolic activity, and support superior growth. However, the four-component OMFA is more efficient in terms of feed required per unit of gain. Thus, the observed reduction in growth rate associated with lactulose supplementation remains unresolved, particularly given that SCFA production should, in theory, facilitate cellular uptake of arginine [56].

## CONCLUSION

This study demonstrated that both the three-component and four-component OMFA exerted beneficial effects on broiler chicken productivity, nutrient digestibility, and gut microbial composition. The four-component OMFA, which included lactulose, significantly improved feed efficiency by reducing feed intake per kilogram of gain by 9.6% and enhanced the accumulation of essential macro- and microelements, such as magnesium, calcium, manganese, cobalt, zinc, and chromium, in muscle tissue. It also modulated the cecal microbiota by promoting the proliferation of *Ruminococcaceae* and suppressing potential pathogens such as *Pseudobdellovibrionaceae*. In contrast, the three-component OMFA without lactulose resulted in the highest weight gain (2.4 kg), increased the digestibility of CF and protein, and elevated the abundance of bacterial families such as *Helicobacteraceae*, *Rikenellaceae*, and *Bacteroidaceae*, although at the cost of slightly higher feed consumption.

The principal strength lies in its integrative approach, which combines zootechnical performance data with detailed microbiome profiling and elemental tissue analysis. This multifactorial assessment allowed for a more comprehensive understanding of how OMFA components interact to influence growth, metabolism, and microbial ecology in broilers. Moreover, the use of high throughput 16S rRNA sequencing provided valuable insights into taxonomic shifts associated with each additive formulation.

However, the study has certain limitations. While microbiome diversity and composition were characterized, functional profiling of microbial communities (e.g., through metagenomics or metabolomics) was not conducted. Additionally, the mechanistic interactions among OMFA components, particularly the potential antagonism between lactulose and other bioactives (e.g., ultrafine SiO_2_ or arginine), remain unclear. The study was also limited to a single breed and management system, which may restrict generalizability.

Future research should explore the temporal dynamics of microbiota shifts in response to OMFA, the metabolic pathways activated by different feed additives, and the histomorphological changes in the intestinal mucosa. Investigating optimal dosing strategies and the use of targeted carriers or encapsulation technologies could further enhance the efficacy and stability of OMFA formulations. Extending the research to include economic evaluations and long-term effects on meat quality and immune competence would provide valuable guidance for practical applications in sustainable poultry production.

## DATA AVAILABILITY

The datasets generated during the present study are available from the corresponding author upon a reasonable request.

## AUTHORS’ CONTRIBUTIONS

EAS: Conceptualization, project administration, and writing – review and editing. DES and API: Investi-gation and writing – original draft. EVY: Methodology. KSN and KVR: Formal analysis and data curation. All authors have read and approved the final manuscript.
